# The Role of Inflammation in the Pathogenesis of Preeclampsia

**DOI:** 10.1155/2020/3864941

**Published:** 2020-10-05

**Authors:** Michał Michalczyk, Aleksander Celewicz, Marta Celewicz, Paula Woźniakowska-Gondek, Rafał Rzepka

**Affiliations:** ^1^Department of Gynecology and Obstetrics, Collegium Medicum, University of Zielona Góra, Zielona Góra, Poland; ^2^Department of Obstetrics and Gynecology, Pomeranian Medical University, Szczecin, Poland

## Abstract

Preeclampsia (PE) affects 5-8% of pregnant women, and it is the major cause of perinatal morbidity and mortality. It is defined as arterial hypertension in women after 20 weeks of gestation which cooccurs with proteinuria (300 mg/d) or as arterial hypertension which is accompanied by one of the following: renal failure, liver dysfunction, hematological or neurological abnormalities, intrauterine growth restriction, or uteroplacental insufficiency. Currently, pathophysiology of preeclampsia poses a considerable challenge for perinatology. Preeclampsia is characterized by excessive and progressive activation of the immune system along with an increase in proinflammatory cytokines and antiangiogenic factors in fetoplacental unit as well as in vascular endothelium in pregnant women. A single, major underlying mechanism of preeclampsia is yet to be identified. This paper discusses the current understanding of the mechanisms which underlie the development of the condition. Some significant factors responsible for PE development include oxidative stress, abnormal concentration and activity in mononuclear phagocytic system, altered levels of angiogenic and antiangiogenic factors, and impaired inflammatory response triggered by inflammasomes. Detailed understanding of pathophysiology of inflammatory process in PE can largely contribute to new, targeted anti-inflammatory therapies that may improve perinatal outcomes in PE patients.

## 1. Introduction

Preeclampsia (PE) affects 5-8% of pregnant women, and it constitutes the major cause of perinatal morbidity and mortality [1]. The International Society for the Study of Hypertension in Pregnancy (ISSHP) defines PE as a combination of arterial hypertension and proteinuria (300 mg/d) present after 20 weeks of gestation or as arterial hypertension which is accompanied by one of the following: renal failure, liver dysfunction, hematological or neurological abnormalities, intrauterine growth restriction (IUGR), or uteroplacental insufficiency [2]. Nowadays, pathophysiology of preeclampsia poses an actual challenge for perinatology [3]. The causes of the condition can be ascribed to excessive maternal systemic inflammatory response to pregnancy. The response is elicited by the activation of innate and adaptive immune systems [4, 5] to the degree which is determined by both environmental and genetic factors [6–11].

In normal pregnancy, the maternal spiral arteries are invaded by extravillous trophoblast cells (EVT) leading to gradual replacement of vascular endothelium. The process is referred to as spiral artery remodeling [12], and when impaired, it leads to placental ischemia and ischemia-reperfusion injury as a result [13]. Failure to sufficient vascular remodeling results in pathological narrowing of spiral arteries and placental blood flow reduction [14], which, as a consequence, leads to tissue ischemia, damaged vascular endothelium, microangiopathic thrombosis, oxidative stress, and inflammatory response [15]. The degree of impairment to trophoblast invasion is also affected by the severity of arterial hypertension in patients with PE [16].

Inflammasomes are high in molecular weight, multimeric, and self-organizing protein complexes of the innate immune system which do not only play a significant role in inflammatory response activation and the release of IL-1*β* and IL-18 but also function like a finely tuned alarm in cellular apoptosis regulation by triggering and enhancing systems in response to stress and/or cellular infections. Following the inflammasome signaling activation, inflammatory processes can potentially promote the development and secretion of proinflammatory cytokines including danger signaling and pyroptotic cell death, i.e., quick inflammation-induced apoptosis.

Contrary to immunosuppression which occurs in normal pregnancy, preeclamptic pregnancy is characterized by excessive immune activation. Th1 cells, NK cells, and self-reactive B cells stimulate the inflammatory response through cytokines activity, which results in an inappropriate trophoblast invasion and impaired spiral artery remodeling in early pregnancy [17, 18]. Uteroplacental underperfusion is therefore the cause of placental ischemia which triggers oxidative-inflammation cascade and increases production of antiangiogenic factors: soluble fms-like tyrosine kinase 1 (sFlt-1) and soluble endoglin (sEng) [19–21].

Preeclampsia is characterized by excessive and progressively increased immune activation with a rise in proinflammatory cytokines and antiangiogenic factors both in the intrauterine environment and maternal endothelium [22], which is the cause of placental dysfunction and maternal systemic complications [23, 24].

## 2. Activation of Response Inflammatory as One of the Causes of PE

The exchange of nutrient and oxygen between maternal and fetal circulatory systems is the crucial role of the placenta. The fetal/maternal exchange takes place at the chorionic villi cellular membrane and intervillous space filled with maternal blood. For normal placental functioning, it is of key importance for placentation to coincide with spiral artery remodeling by extravillous trophoblast [25]. A vital role here is played by reactive oxygen species (ROS) as signaling pathways necessary for proper placentation in normal pregnancy. Moreover, low oxygen level in early pregnancy is also a significant factor which stimulates placental angiogenesis [26, 27].

In the first trimester of gestation, the fetus develops in low oxygen concentration, which promotes trophoblast cells proliferation. During placentation in the first weeks of gestation, the uterine arteries undergo a distinctive transformation to become vessels of high volume and low resistance capacity. This specific and unique process results in elevated intervillous oxygen level during the first trimester, which entails a significant enhancement to oxygen exchange at the maternal-fetal interface to satisfy the needs of the developing fetus [13, 28, 29]. Additionally, impaired trophoblast invasion causing temporary ischemia with subsequent reperfusion creates contributory conditions for oxidative stress, which appears to lead to consequent endothelial damage and inflammatory activation [30–32]. Such a pathological condition induces systemic inflammatory response by activation of endothelial cells and other cell types, a release of proinflammatory cytokines, and cellular debris shed by syncytiotrophoblast (STB) [33, 34]. A large number of studies have already determined that maternal immune malfunction, particularly within innate immunity compartment, which also contributes to the activation of the immune response involved in pathogenesis of PE. What plays a significant role here are the changes to mononuclear phagocyte system [35]. Macrophages are vital for tissue homeostasis; they regulate inflammatory process and are key regulators of tissue repair.

Systemic inflammatory response is characteristic of all pregnancies; however, in pregnancies complicated with PE inflammatory, response reaches extreme intensity [36]. This is expressed by abnormally upregulated immune reactions to the activation of innate immune system and other proinflammatory factors [17]. Consequently, inappropriate trophoblast invasion in myometrium and insufficient spiral arteries remodeling result in placental ischemia. Furthermore, the ensuing oxidative stress is enhanced by excessive release of placental factors: syncytiotrophoblast-derived extracellular vesicles (SEDVs), sFlt-1, and vascular endothelial growth factor (VEGF) that enter maternal circulation, which also contribute to arterial hypertension [37, 38]. These angiogenic factors are also potent mediators of inflammatory response, and they augment inflammation symptoms in PE patients [39]. Cytotrophoblast secreting interleukins 1*β*, 2, 4, 6, 8, 10, 12, and 18, transforming growth factor *β*1 (TGF*β*1), IFN-*γ*-inducibleprotein10/IP-10, tumor necrosis factor (TNF-*α*), interferon *γ* (IFN-*γ*), monocyte chemoattractant protein-1 (MCP-1), intercellular adhesion molecule- (ICAM-) 1, and vascular cell adhesion molecule- (VCAM-) 1 also contributes to PE development [17, 40–43]. Certain cytokines (IL-1*β* and IL-18) also affect maternal vascular endothelium and cause its dysfunction [43]. Moreover, by direct or indirect activation of other inflammatory pathways, these cytokines can exacerbate clinical symptoms of PE. The factors which augment the inflammatory response in syncytiotrophoblast also include cholesterol and uric acid [44]. Molecular mechanisms regulating human placental inflammatory response involve the so-called inflammasomes which activate protease, caspase-1 (K1), leading to proinflammatory cytokines IL-1*β* and IL-18 activation and secretion of interferon *γ*, and finally IL-6 [45]. Given that as yet no effective PE therapy has been developed and delivery of the placenta remains the only definitive treatment, thorough investigation of molecular pathways accountable for the cause and severity of the condition seems to be a promising way to design PE prevention and/or treatment methods.

## 3. Oxidative Stress as the Cause of Inflammatory Response in PE

Activation of the oxidative stress is the condition that must be met for the physiological pregnancy to develop [46]. Oxidative stress involves an imbalance between reactive oxygen species and tissue antioxidant defense system [47–49].

Nitric oxide (NO) regulates vascular tone in order to increase uterine blood flow, and it modulates vasodilation which depends on the endothelial functioning and is also regulated by pregnancy-induced estrogen increase. Nitric oxide is released from endothelial cells, and its two-way activity includes relaxation of blood vessel walls and blood anticoagulation. Endothelial nitric oxide synthase isoform (eNOS) affects endothelium by reducing vascular wall tension, and it inhibits platelet and leukocyte adhesion to the vascular endothelium, which plays a key role in attenuation of inflammatory response. On the other hand, the inflammation-induced inducible nitric oxide synthase (iNOS) generates excessive amounts of NO. This inflammation-related endothelial dysfunction is considered one of potential causes of PE [50, 51].

The most significant elements of defense against ROS include enzymatic antioxidants: superoxide dismutase (SOD), glutathione peroxidase (GPx), and catalase (CAT). Antioxidant defense system also involves vitamin C, E, *α* tocopherol, *β*-carotene, ubiquinone, carotenoids, and glutathione [52]. In the first trimester of gestation, placental tissue is characterized by low content and activity of enzymatic antioxidants such as CAT, GPx, Cu/Zn, and Mn-SOD, which makes trophoblasts particularly susceptible to oxidative damage [53]. Thus, at the beginning of the first trimester, when the intervillous blood oxygen level increases approx. 3-fold due to maternal blood flow to the placenta, reactive oxygen levels are observed to increase rapidly [54, 55].

The major role that ROS plays in PE pathogenesis concentrates on stimulating the secretion of proinflammatory cytokines, chemokines, and cellular debris from apoptotic changes to syncytiotrophoblast [56]. Oxidative stress is also accountable for the activation of NLRP3 inflammasome, caspase-1, and, consequently, IL-1*β* release [56, 57]. PE can be characterized as an inflammatory response to placental ischemia and its subsequent reperfusion [53]. Reperfusion-induced placental damage coincides with pathological inflammatory response, which leads to aggravated systemic inflammatory responses and tissue damage by ROS. Scientific evidence clearly indicates that reduced placental blood flow caused by pathological trophoblastic invasion and abnormal angiogenesis induces placental oxidative stress which results in vascular inflammation and endothelial dysfunction [58]. Increased cellular exposition to ROS causes protein carboxylation, lipid peroxidation, and DNA oxidation. These changes are typically found in preeclamptic placentas. When intracellular ROS production is on the increase, interaction between NO and ROS results in formation of peroxynitrite (ONOO^−^) which, in turn, causes eNOS inactivation. In the face of the loss of enzymatic activity, tissue homeostasis is compromised, and oxidative damage to the placenta occurs thereby inducing inflammatory response and a release of large amounts of SEDVs. Activation of the inflammatory response triggered by oxidative stress plays a key role in the etiology of PE [58].

## 4. Monocytes and Macrophages as the Cause of Inflammatory Response in PE

Normal functioning of the mononuclear phagocytic system is the crucial element of human innate immunity. Scientific data suggest that a disruption to the immune response in preeclamptic pregnancy is caused by disturbance to phagocytic system activity [35]. Monocytes fall into three major groups: classical, intermediate, and nonclassical. Tissue macrophages can be divided into proinflammatory (M1) and anti-inflammatory (M2). Monocyte differentiation is controlled by cytokines [35]. It has not been decisively determined yet which monocyte subset is most predisposed to differentiate to M1 or M2 macrophages, and whether identical monocytes can undergo tissue-dependent differentiation to M1 and/or M2. Whether selective exhaustion of a selected monocyte subset affects the tissue composition of macrophage population is yet to be determined too.

What mechanisms specifically underlie monocytes activation in pregnancy remain unknown. Syncytiotrophoblast is considered to play the critical role in the process [59, 60]. Quantitative and qualitative profiles of circulating monocytes reflect the severity of preeclamptic pregnancy. The monocyte count and monocyte/lymphocyte ratio have been demonstrated to be higher in preeclamptic women than in normal pregnancies [61, 62]. In preeclamptic pregnancies, reduced levels of anti-inflammatory (classical) monocytes and significantly elevated levels of proinflammatory (intermediate and nonclassical) monocytes are observed in comparison with normal pregnancies [63–65]. Ma et al. analyzed proinflammatory cytokines in blood serum of pregnant women with PE and estimated the percentage of monocytes positive for M1 and M2 markers. CD14+CD11c+CD163-(M1) monocytes in PE women were found to be significantly higher, which correlated with elevated levels of proinflammatory mediators: IL-1*β*, IL-6, and MCP-1 [66]. As nonclassical subpopulation becomes more dominant and numerous, the systemic inflammatory response is augmented in preeclamptic patients. The inflammatory response enhancement is caused by extracellular factors and cytokines which activate monocytes [67]. In the future, monocyte count and flow cytometry monocyte phenotyping may play a significant role in predicting disease progression in PE.

Proper decidual balance between pro- and anti-inflammatory macrophages is vital for normal pregnancy development. The increase in nonclassical macrophages subpopulation can affect tissue macrophages system in the endometrium and can be accountable for disturbed placentation in preeclamptic pregnancy [67]. Macrophage polarization into M2 macrophages, physiologically occurring in the second trimester, is believed to be inhibited in preeclamptic pregnancies [21]. Consequently, there is no suppression to M1 macrophages activity, which increases the production of proinflammatory cytokines IFN-*γ*, TNF-*α*, and IL-6 and decreases IL-4 and IL-10 levels [21, 68].

The shift in macrophage differentiation from M2 to M1 is ascribed to high levels of proinflammatory and low levels of anti-inflammatory cytokines in preeclamptic placental tissue [69, 70]. Apart from cytokines, what has an impact on macrophage differentiation is the so-called cell axis. A large number of studies have reported that it is the placental mesenchymal stem cells that play a key role in macrophage differentiation into one of the subpopulations, M1 or M2. They are also capable of selective activation of macrophages [71, 72]. Wang et al. determined an important function of hyaluronate in normal pregnancy development. High hyaluronate levels were found to stimulate macrophage polarization to M2 subtype and regulate cytokine production (i.e., IL-10) by decidual macrophages [73].

## 5. Inflammasomes as the Cause of Inflammatory Response in PE

Inflammasomes are cytosolic multiprotein complexes composed of pattern recognition receptor (PRR), apoptosis-associated speck-like protein containing a caspase recruitment domain (ASC), and proinflammatory caspase-1 [74]. The inflammasome recognition receptor is responsible for the response to microbe-derived (viral, bacterial) pathogens: pathogen-associated molecular pattern (PAMP), the body's own cells affected by stress: stress-associated molecular pattern (SAMP), and the cells from damaged tissue: damage-associated molecular pattern (DAMP) [75, 76]. The activated receptor leads to the consequent inflammasome self-oligomerization and caspase-1 activation [77]. This initiates low-level inflammatory response causing IL-1*β* and IL-18 release [78–80] and pyroptotic cell death (inflammation-induced apoptosis) [81, 82]. Originally, inflammasomes were considered specific to innate immunity response [83]; however, recent analyses have reported that they are also engaged in the promotion of adaptive immunity [84, 85]. Cellular potential for inflammatory signaling is largely dependent on PRR expression on the cell surface. Two major classes of PRR families include Toll-like receptors (TLRs) and Nod-like receptors (NLRs) [86].

TLRs are transmembrane receptors which recognize PAMPs and DAMPs outside of the cell and in intracellular endosomes. To date, ten TLRs have been identified and described. Each of these receptors is stimulated by their specific ligands thereby triggering signaling cascades in response to infection caused by Gram (+)/Gram (-) bacteria or RNA viruses [87, 88].

NLRs belong to the cytosolic PRRs and constitute a system of intracellular sensors of DAMPs or PAMPs. The inflammatory response activated by NRLs is thus stimulated and sustained by endogenous “danger” signals. Numerous NLRs and NLR-dependent inflammasomes have been identified so far, and these include pyrin domain-containing proteins (NLRP1, NLRP3), NLR-family caspase activators, caspase activation and recruitment domain (CARD), domain-containing protein-4 (NLRC4), and apoptosis-associated speck-like protein containing a CARD (ASC) [89].

**Figure 1:**
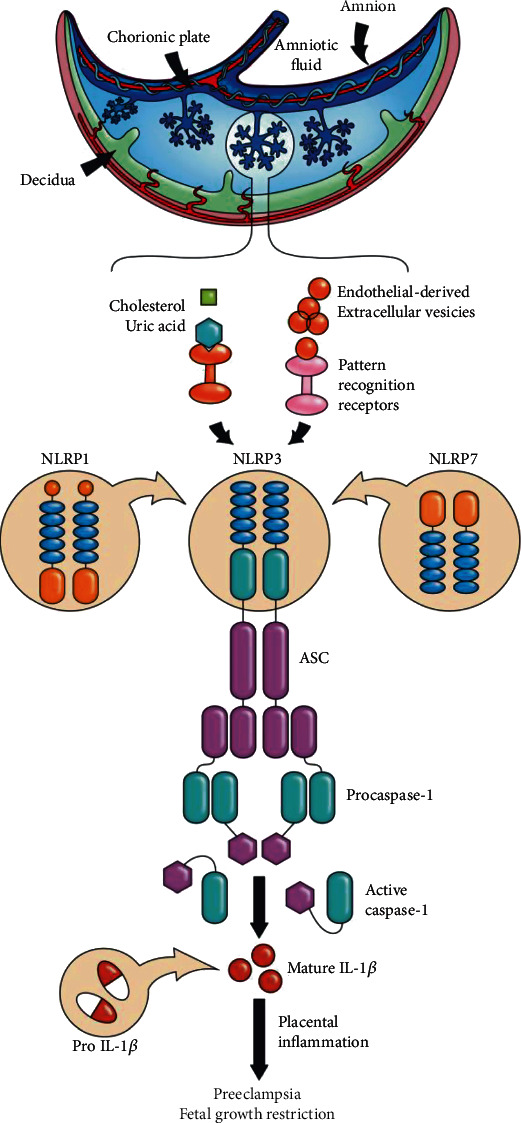
Inflammasomes in placental inflammation. Endothelial-derived extracellular vesicles and/or alarmins (e.g., cholesterol or uric acid) can activate the NLRP3, NLRP1, and NLRP7 inflammasomes in the placenta, leading to the processing and release of active caspase-1 and mature IL-1*β*. The resulting inflammation may lead to placental diseases such as preeclampsia and fetal growth restriction.

Since inflammasome components are expressed on placental cells, recent studies report that inflammasomes are actively involved in inflammatory response related to placental dysfunction in PE. Mulla et al. [90] and Xie et al. [88] proved that NRLP3 activation in trophoblasts and peripheral blood plays an important role in the pathogenesis of PE. Moreover, enhanced expression of NLRP1 and NLRP3 has been demonstrated in peripheral monocytes from preeclamptic women [91, 92]. Additionally, women with PE demonstrate an elevated level of total cholesterol and uric acid which both belong to host-derived damage-associated molecular patterns (DAMPs)—endogenous alarmins [93, 94]. This, in turn, can lead to the NLRP3 inflammasome activation in syncytiotrophoblasts. The NLRP3 inflammasome is the most widely investigated and therefore the best-characterized of all the inflammasomes. It is distinguished by its two major features: its activation can be triggered by a variety unrelated factors (including PAMPs, DAMPs, and alarmins) [95, 96], and it is highly expressed on the cells of innate immune system (macrophages, neutrophils, and dendritic cells) in many tissues [97, 98]. The NLRP3 inflammasome activation can be described as a two-signal model. The first so-called priming signal is initiated by inflammatory stimuli which affect transmembrane PRRs (TLRs) and activate the NF-*κ*B pathway, leading to upregulation of pro-IL-1*β* and NLRP3 protein levels [99]. The second signal involves simultaneous signaling pathways after PAMP or DAMP recognition: they initiate assembly of the NLRP3 inflammasome complex and activate procaspase-1 into its cleaved form, which results in the release of IL-1*β* and IL-18 (Figure 1) [75].

Several molecular mechanisms are triggered by NLRP3 activation, and these, among others, include potassium ion efflux, lysosomal rupture, mitochondrial dysfunction, calcium influx, and decrease of intracellular cAMP [100–102]. A well-established mechanism of NLRP3 inflammasome activation is a decrease in the intracellular potassium concentration. It confirms the assumption that numerous microbiological and endogenous signals can activate inflammasomes by reducing the level of cytosolic potassium [103]. However, signaling pathways of inflammasome activation which are independent of changes to cytosolic potassium concentration have also been described [104]. The NRLP3 inflammasome can also be activated in a caspase-11-mediated noncanonical pathway. The signaling pathway was first described in mice infected with *Escherichia coli*, *Citrobacter rodentium*, and *Vibrio cholerae* (caspase-11 homologs in humans are caspase-4 and caspase-5) [105–107]. Similarly to the canonical pathway results, noncanonical activation pathway leads to caspase-1 cleavage resulting in IL-1*β* and IL-18 release. However, caspase-11 sensing and binding with LPS are typical to the noncanonical pathway only [108]. Active caspase-11 also cleaves gasdermin D (GSDMD), which allows the N-terminal domain of GSDMD to form pores in the plasma membrane thereby triggering pyroptosis, proinflammatory form of cell death, linked to caspase-11 activation, and the release of IL-1*β* and IL-18 [109, 110]. Inflammasomes play one of the critical roles in the process of the host protection against pathogens, and they are actively involved in the immunoregulation, which is of key importance for the systemic homeostasis [111]. Therefore, any pathological interference in inflammasomes activation may augment placental inflammatory response thereby inducing clinical and laboratory symptoms related to PE.

## 6. Conclusions

Over the recent years, our understanding of the pathophysiological background of PE has been enriched considerably. Preeclampsia involves a chronic activation of maternal immune system which is demonstrated by elevated proinflammatory cytokine levels and simultaneously reduced influence of immunoregulatory factors. The imbalance is promoted by prolonged inflammatory response in pregnancies complicated with PE. To date, a single and conclusive mechanism underlying preeclampsia has not been identified yet. The analyses to determine the cause of the immune imbalance leading to enhanced systemic inflammatory response which occurs in PE appear to be an intriguing aim for further research which may contribute to the identification of new targets for PE therapies. More thorough understanding of the pathophysiology of inflammatory process in preeclampsia can largely contribute to the design of new, targeted anti-inflammatory therapies. The examples of such potential include the use of highly selective NRLP-3—inflammasome inhibitor MCC950. This diarylsulfonylurea-containing compound blocks the release of proinflammatory IL-1*β* by preventing oligomerization of the inflammasome adaptor protein ASC. Flow cytometry monocyte phenotyping could also be applied in therapies for PE. Finally, bearing in mind the oxidative stress underlying the inflammatory response activation in PE, antioxidant therapy could also provide a promising solution. In conclusion, the findings of further research into the subject will have a key impact on the development of a targeted therapy which can improve the perinatal outcomes in women affected with preeclampsia.

## Data Availability

No data were used to support this study.
